# Consumer Reported Care Deferrals Due to the COVID-19 Pandemic, and the Role and Potential of Telemedicine: Cross-Sectional Analysis

**DOI:** 10.2196/21607

**Published:** 2020-09-14

**Authors:** Adam Atherly, Eline Van Den Broek-Altenburg, Victoria Hart, Kelsey Gleason, Jan Carney

**Affiliations:** 1 Center for Health Services Research Larner College of Medicine University of Vermont Burlington, VT United States; 2 Department of Radiology Larner College of Medicine University of Vermont Burlington, VT United States; 3 Department of Medicine Larner College of Medicine University of Vermont Burlington, VT United States

**Keywords:** COVID-19, telemedicine, deferred care, mental health, alternative, health effect, viability

## Abstract

**Background:**

The COVID-19 pandemic forced many health systems to proactively reduce care delivery to prepare for an expected surge in hospitalizations. There have been concerns that care deferral may have negative health effects, but it is hoped that telemedicine can provide a viable alternative.

**Objective:**

This study aimed to understand what type of health care services were being deferred during the COVID-19 pandemic lockdown, the role played by telemedicine to fill in care gaps, and changes in attitudes toward telemedicine.

**Methods:**

We conducted a cross-sectional analysis of survey responses from 1694 primary care patients in a mid-sized northeastern city. Our main outcomes were use of telemedicine and reports of care deferral during the shutdown.

**Results:**

Deferred care was widespread—48% (n=812) of respondents deferred care—but it was largely for preventive services, particularly dental and primary care, and did not cause concerns about negative health effects. In total, 30.2% (n=242) of those who delayed care were concerned about health effects, with needs centered around orthopedics and surgery. Telemedicine was viewed more positively than prior to the pandemic; it was seen as a viable option to deliver deferred care, particularly by respondents who were over 65 years of age, female, and college educated. Mental health services stood out for having high levels of deferred care.

**Conclusions:**

Temporary health system shutdowns will give rise to deferred care. However, much of the deferrals will be for preventive services. The effect of this on patient health can be moderated by prioritizing surgical and orthopedic services and delivering other services through telemedicine. Having telemedicine as an option is particularly crucial for mental health services.

## Introduction

The COVID-19 pandemic led to an unprecedented worldwide economic and health system shutdown. COVID-19 is caused by the novel SARS-CoV-2, which first emerged in December 2019 in Wuhan, China [1]. As of July 1, 2020, COVID-19 cases have been reported in more than 200 countries with more than half a million confirmed deaths [2]. The first case in Vermont (the region of this study) was reported on March 7, 2020, and the number of cases in Vermont peaked in late April 2020.

One effect of the pandemic was a widespread temporary shutdown of health care services to prepare the health care system for an expected increase in COVID-19 cases. One unintended consequence of this temporary shutdown is that many types of health care services were deferred until after the “peak” of COVID-19 cases [3-5]. A recent report found that nearly half of all adults reported that either they or someone in their household deferred medical care due to the coronavirus outbreak [6].

As health care systems begin their gradual reopening, the backlog of cases is beginning to be resolved. Yet there is controversy about which services should be reopened and prioritized, with dental care services being a particular flashpoint [7,8]. Health care leaders are trying to balance patient needs and the risk of COVID-19 infection as they consider what health care service lines should be prioritized. Yet little is known about what proportion of patients had deferred care during the pandemic or what types of services are most needed from the patient perspective.

Another unintended consequence of the shutdown is the sudden prominence of telemedicine. Telemedicine is not new, but the ability to deliver care remotely without asking patients to increase their risk of infection has brought telemedicine to new prominence during the pandemic. One of the first administrative acts by the Trump administration was to temporarily relax restrictions on telemedicine delivery [9]. Beginning in early March 2020, Medicare began paying for telehealth visits for a broader set of services, locations, modalities, and professions, as well as waiving requirements such as having an established patient-provider relationship [10]. These changes were intended to allow the emergency expansion of telehealth services during the height of the pandemic from April to June 2020.

One approach that can safely deliver nonemergency care can be telemedicine use for routine care. Much of the nonemergency care that occurred during the peak of the crisis was delivered via telemedicine. This created a sudden introduction to telemedicine for both patients and (some) health care professionals. It has been suggested that the pandemic may permanently change the role of telemedicine in America [11], yet whether views on telemedicine have actually changed is unknown.

Our objective was to present the findings from a recent survey of primary care patients in Vermont about both deferred care and telemedicine. We describe for what services care was deferred and where patient concerns about deferred care are highest. We then explore attitudes toward telemedicine, whether those attitudes have changed, and how telemedicine may be used for deferred care if there is another health care system shutdown. Our data were drawn at a unique time to measure care deferral—while it was happening. The results of this study can provide guidance on what is likely to happen in the future if a similar lockdown of health services occurs.

## Methods

The study design is a cross-sectional analysis of the primary care population of the greater Burlington area in Vermont. A random sample of 12,000 individuals over 18 years with at least one primary care visit during the preceding 3 years was drawn from University of Vermont Medical Center patients, stratified by age cohort. Within each 10-year age cohort, a random number generator was used to select the sample. The unit of observation was the individual patient.

Individuals were contacted in two waves between April 30 and May 13, 2020, and asked to consent to participate in the survey. All individuals were provided with an opportunity to opt out of the survey. A total of three follow-up reminders was sent. The survey took an estimated 16-20 minutes to complete and covered a number of different topic areas. These topics included sociodemographic data (eg, age, gender, income, household type and composition, etc), general health status and risk factors (eg, pre-existing health conditions; smoking; COVID-19 symptoms, testing, and diagnosis), exposure to COVID-19 and prevention actions undertaken by the individual, economic impact of the shutdown, beliefs about and preferences for public interventions, telemedicine, and delayed care.

Key variables for our analysis on deferred care included whether the respondent had deferred care due to COVID-19 (yes/no) (“Have you had to defer needed health care because of the COVID-19 outbreak and response?”). For those who answered yes to deferring care, we asked how concerned they were that the delay would harm their health (on a 5-point Likert scale, from *very concerned* to *very unconcerned*); we also asked them to specify the type of health service deferred (eg, primary care, dental, oncology, etc). For telemedicine, individuals were asked if they had ever used telemedicine (yes/no), whether they were more or less likely to use telemedicine now versus before the pandemic, and whether they would consider using telemedicine for deferred care.

Key control variables include age (in categories), income (in categories), gender, education (college: yes/no), and presence of chronic illnesses (yes/no from a list including conditions identified by the Centers for Disease Control and Prevention as increasing the risk of COVID-19 complications) [12]. We also included variables indicating whether the person had lost their job due to COVID-19. For those who had an income reduction due to COVID-19, we asked by how much their income had declined.

Means and frequencies from the data were calculated. For our analysis of reasons for deferred care, the denominator is individuals who reported deferring care. For the telemedicine analysis, the denominator is the entire sample. For dichotomous variables (*increased interest in telemedicine* and *willingness to use telemedicine for deferred care*), we performed a multivariate analysis using a logit model, with the coefficients calculated as odds ratios. Variables were considered to be statistically significant with a *P* value <.05.

The study was approved by the Institutional Review Board of the University of Vermont. Participants were not compensated for study participation.

## Results

The initial sample comprised 12,000 individuals. Of these, 11,700 (98%) had a functioning email address. We had a total of 2275 responses (19.4%), and 1961 (16.3%) individuals both read the consent form and agreed to participate. Of these, 1694 (14.1%) people completed the survey.

Overall, 48% of the sample (803 out of 1694 completed responses) reported deferring care due to COVID-19. Of these 803 individuals who reported deferred care, 78% (n=626) reported that the care was deferred due to a cancellation of the appointment by the health care provider rather than due to a decision by the respondent, while in 22% (n=177) of cases the respondent decided to cancel. The sample was relatively evenly distributed by age (Table 1).

**Table 1:** Descriptive statistics from the sample of 1681 observations showing frequencies and percentages in different categories.

Variable		Respondents, n (%)
**Age group (years)**		
	18-24	62 (3.69)
	25-34	285 (16.95)
	35-44	327 (19.45)
	45-55	365 (21.71)
	55-64	412 (24.51)
	65-75	230 (13.68)
**Income ($ USD)**		
	Prefer not to answer	100 (5.95)
	$1000-$25,000	50 (2.97)
	$25,001-$50,000	145 (8.63)
	$50,001-$75,000	241 (14.34)
	$75,001-$100,000	338 (20.11)
	>$100,001	807 (48.01)
**Education**		
	Less than college	401 (23.85)
	College graduate	1280 (76.15)
**Location**		
	Urban	501 (29.8)
	Semiurban/suburban	841 (50.03)
	Rural	339 (20.17)
**Live alone**		
	No	1465 (87.15)
	Yes	216 (12.85)
**Sex**		
	Male	691 (41.11)
	Female	990 (58.89)
**Presence of chronic illness**		
	No	765 (45.51)
	Yes	916 (54.49)

The top reported service line for deferred care was dental services, with 27% (n=219) of the sample reporting deferred care for dental services (Table 2). Dental care was followed by primary care (n=183, 23%) and other services (n=140, 18%). Considerably fewer respondents reported deferring care for orthopedics (n=65, 8%), women’s health (n=56, 7%), and radiology/imaging (n=45, 6%).

**Table 2:** Delayed care by service line among 803 survey respondents in Vermont between April 30 and May 13, 2020, who reported deferring care due to the COVID-19 pandemic medical services shutdown.

Health care service	Respondents, n (%)
Dental services	219 (27)
Primary care	183 (23)
Other	140 (18)
Orthopedics	65 (8)
Women’s health/obstetrics and gynecology	56 (7)
Radiology/imaging	45 (6)
Surgery	22 (3)
Internal medicine	20 (2)
Mental health	17 (2)
Cancer	10 (1)
Neurology	10 (1)
Pediatrics	8 (1)
Cardiovascular	7 (1)

Overall, 68% (n=546) of those who deferred care reported that the purpose of the intended services was preventive care (Table 3). Only 38% (n=305) reported deferring care for existing problems and 29% for newly emergent problems (n=233), which varied across service type. Dental services had the highest level of deferred care overall. Of those who deferred dental care, it had the highest level of deferred care for preventive services (n=184, 84%) and the lowest level of deferred care for ongoing (n=45, 21%) and newly emergent problems (n=37, 17%). In contrast, of those who deferred care for surgery, 68% (n=15) were for newly emergent issues and 45% (n=10) for ongoing issues. Orthopedics also stands out for high levels of deferred care for newly emergent (n=33, 51%) and ongoing (n=41, 63%) care. Note that these percentages do not necessarily add to 100% because respondents could check multiple categories.

**Table 3:** Reasons for delayed care, by service line and problem type, among 803 survey respondents in Vermont between April 30 and May 13, 2020, who reported deferring care due to the COVID-19 pandemic medical services shutdown.

Health care service	New developed problem, n (%)	Care for ongoing problems, n (%)	Preventive care, n (%)
Dental services	37 (17)	45 (21)	184 (84)
Primary care	52 (29)	56 (30)	137 (75)
Orthopedics	33 (51)	41 (63)	23 (35)
Women’s health/obstetrics and gynecology	18 (32)	21 (38)	40 (71)
Radiology/imaging	14 (30)	14 (30)	33 (74)
Surgery	15 (68)	10 (45)	6 (27)
Internal medicine	8 (40)	10 (50)	13 (65)
Mental health	4 (24)	13 (76)	7 (41)
Cardiovascular	3 (43)	4 (57)	6 (86)
Other	38 (27)	78 (55)	80 (57)
Total	229 (29)	309 (38)	547 (68)

Overall, most respondents who deferred care reported relatively low levels of concern about the health effects of the delay (Table 4). Overall, only 5.1% (n=41) of those who deferred care were *very concerned* about the health effect of the delay, 25.0% (n=201) were *concerned*, 30% (n=241) were *neutral* about the effect, and 40% (n=320) were either *unconcerned* or *very unconcerned*. However, the level of concern reported by the survey respondents also varied across service lines. The highest level of concern was for mental health services (*very concerned* / *concerned*: n=10, 59%), followed by surgery (n=12, 55%) and orthopedics (n=32, 50%). The lowest level of concern was for primary care (n=35, 19%) and dental services (n=60, 28%), which were also the most common services for which care was deferred.

**Table 4:** Level of concern about care delays overall and by service line among 803 survey respondents in Vermont between April 30 and May 13, 2020, who reported deferring care due to the COVID-19 pandemic medical services shutdown.

Health care service	Level of concern, n (%)				
	Very concerned	Concerned	Neutral	Unconcerned	Very unconcerned
Dental services	4 (1.8)	56 (25.8)	56 (24.9)	79 (36.4)	24 (11.1)
Primary care	7 (3.3)	28 (15.4)	64 (35.2)	62 (34.1)	22 (12.1)
Orthopedics	11 (16.9)	21 (32.3)	15 (23.1)	11 (16.9)	7 (10.8)
Women’s health/obstetrics and gynecology	4 (7.1)	11 (19.6)	23 (41.1)	10 (17.9)	8 (14.3)
Radiology/imaging	5 (11.6)	11 (23.3)	17 (37.2)	10 (23.3)	2 (4.7)
Surgery	1 (4.6)	11 (50.0)	8 (36.4)	0 (0.0)	2 (9.1)
Internal medicine	1 (5.0)	7 (35.0)	5 (25.0)	2 (10.0)	5 (25.0)
Mental health	1 (5.9)	9 (52.9)	3 (17.7)	3 (17.7)	1 (5.9)
Other	5 (3.6)	35 (25.0)	39 (27.9)	46 (32.9)	15 (10.7)
Total	42 (5.1)	200 (25.0)	242 (30.0)	229 (28.7)	89 (11.2)

Turning to telemedicine, our analysis now includes the entire sample (N=1861). Overall, a minority of the sample had used telemedicine (n=837, 45%), but a strong majority reported that they were more likely to use telemedicine now than before the pandemic (n=1470, 79%) (Table 5). A majority was willing to use it for deferred care (n=1359, 73%), but those respondents were much smaller among individuals who actually deferred care. Among those who deferred care and were concerned about the health effect of the deferral, 59% (n=189) were willing to use telemedicine for the services. Among those who deferred care and were not concerned about the health effect, only 54% (n=261) were willing to use telemedicine to resolve the problem.

**Table 5:** Experience with and willingness to use telemedicine among 1861 survey respondents in Vermont between April 30 and May 13, 2020.

Question		Respondents, n (%)
**Have you ever used telemedicine for health care?**		
	Yes	760 (46)
	No	921 (55)
**Are you more likely to use telemedicine now than before the pandemic?**		
	Yes	1332 (79)
	No	332 (20)
**Would you consider using telemedicine for deferred care?**		
	Yes	1226 (73)
	No	433 (26)
**For those with deferred care and are concerned about health effects: would you use telemedicine for deferred care?**		
	Yes (concerned about health effects)	130 (59)
	Yes (not concerned about health effects)	331 (54)
	No (concerned about health effects)	110 (40)
	No (not concerned about health effects)	226 (45)

The logit analysis of factors explaining factors associated with increases in willingness to use telemedicine tells a similar story (Table 6). The odds ratio (OR) for persons willing to use telemedicine who were *very concerned*/*concerned* about the health effect of deferred care due to the pandemic was 0.34 *(P*=.02) compared to those who were *neutral* or *unconcerned*/*very unconcerned*. However, college graduates (OR 2.15, *P*<.001) and females *(*OR 1.73, *P*<.001) were more likely to use telemedicine (reference groups: noncollege graduates and males), as were those with a chronic illness (OR 1.4*, P*=.009) (reference group: no chronic illness). The largest age effect was in the oldest population (persons ≥65 years: OR 2.29, *P*=.02) (reference group: <25 years). By service line, mental health was the service with the largest increase, although the effect was not statistically significant *(P*=.12).

**Table 6:** Logit regression explaining factors predicting increased willingness to use telemedicine among 1861 survey respondents in Vermont between April 30 and May 13, 2020, with coefficients representing percentage point increases.

Variable			Odds Ratio		SE		z		*P* value		95% CI
Had deferred care			0.341		0.251		–1.460		.143		0.081-1.439
Concerned / very concerned about deferred care			0.629		0.123		–2.370		.018		0.429-0.922
**Age group (years) (reference: 18-24 years)**											
	25-34	1.439		0.474		1.100		.269		0.754-2.744	
	35-44	1.732		0.576		1.650		.099		0.902-3.325	
	45-54	1.571		0.518		1.370		.171		0.823-2.998	
	55-64	1.589		0.513		1.440		.151		0.845-2.990	
	≥65	2.290		0.814		2.330		.020		1.141-4.595	
**Income (reference:** **>$100,001)**											
	Prefer not to answer	0.375		0.091		–4.030		<.001		0.232-0.604	
	$1000-$25,000	1.017		0.386		0.040		.965		0.484-2.138	
	$25,001-$50,000	1.251		0.332		0.840		.400		0.743-2.104	
	$50,001-$75,000	0.994		0.205		–0.030		.976		0.664-1.488	
	$75,001-$100,000	0.716		0.119		–2.010		.045		0.517-0.992	
College graduate			2.146		0.309		5.300		<.001		1.618-2.846
**Income decline (reference: no income decline)**											
	Less than 25%	1.069		0.196		0.360		.718		0.746-1.531	
	50%	0.816		0.200		–0.830		.409		0.505-1.321	
	75%	0.845		0.372		–0.380		.703		0.357-2.003	
	100% (I have lost all my income)	1.446		0.505		1.060		.291		0.729-2.866	
Currently unemployed			0.871		0.171		–0.700		.482		0.594-1.279
**Location (reference: urban area)**											
	Semiurban/suburban	1.203		0.176		1.270		.205		0.904-1.602	
	Rural	1.287		0.235		1.380		.168		0.899-1.840	
Sex: female			1.727		0.225		4.200		<.001		1.339-2.229
Live alone			0.748		0.144		–1.510		.131		0.513-1.091
Have chronic illness			1.400		0.181		2.600		.009		1.086-1.804
**Deferred care in**											
	Orthopedics	2.810		2.230		1.300		.193		0.593-13.309	
	Mental health	4.647		4.561		1.570		.117		0.679-31.812	
	Neurology	1.455		1.489		0.370		.714		0.196-10.808	
	Pediatrics	0.963		1.004		–0.040		.971		0.125-7.439	
	Radiology/imaging	2.600		2.143		1.160		.247		0.517-13.083	
	Surgery	3.534		3.314		1.350		.178		0.562-22.211	
	Primary care	2.688		2.016		1.320		.188		0.618-11.692	
	Cancer	2.540		2.754		0.860		.390		0.303-21.268	
	Cardiovascular	4.071		5.400		1.060		.290		0.303-54.792	
	Internal medicine	1.940		1.729		0.740		.457		0.338-11.132	
	Women’s health/obstetrics and gynecology	2.562		2.067		1.170		.244		0.527-12.458	
	Dental services	2.372		1.768		1.160		.247		0.550-10.223	
	Other	3.830		2.934		1.750		.080		0.853-17.188	

These results are consistent with factors associated with a willingness to use telemedicine for deferred care (Table 7). In this model, having actually deferred care has a strong negative association with willingness to use telemedicine for deferred care (b=0.18, *P*=.02), but the level of concern was not significantly related. This model again shows a strong age effect, with the 45-55, 55-64, and ≥65 age groups all showing ORs significantly greater than 1 versus the reference group (<25 years), with the largest coefficient observed in the ≥65 years group. College graduates, females, and persons with chronic illnesses again had a higher odds, although some of the coefficients were only marginally significant. Location (suburban) was statistically significant (OR 1.42, *P*=.02) and positive compared to urban although rural was not.

**Table 7:** Logit regression explaining factors predicting willingness to use telemedicine for deferred care among 1861 survey respondents in Vermont between April 30 and May 13, 2020 with coefficients representing percentage point increases.

Variable		Coefficient	SE	z	*P* value	95% CI
Had deferred care		0.183	0.135	–2.290	.022	0.043-0.781
Concerned / very concerned about deferred care		0.856	0.144	–0.930	.353	0.616-1.189
**Age group (years) (reference: 18-24 years)**						
	25-34	1.211	0.400	0.580	.562	0.634-2.312
	35-44	2.048	0.682	2.150	.031	1.066-3.934
	45-54	2.241	0.747	2.420	.015	1.166-4.306
	55-64	2.328	0.762	2.580	.010	1.226-4.420
	≥65	2.802	0.989	2.920	.004	1.403-5.596
**Income (reference:** **>$100,001)**						
	Prefer not to answer	0.688	0.182	–1.410	.158	0.409-1.156
	$1000-$25,000	1.114	0.441	0.270	.785	0.513-2.422
	$25,001-$50,000	0.952	0.232	–0.200	.840	0.591-1.535
	$50,001-$75,000	0.868	0.166	–0.740	.458	0.597-1.262
	$75,001-$100,000	0.960	0.159	–0.250	.806	0.694-1.328
College graduate		1.307	0.196	1.790	.073	0.975-1.753
**Income decline (reference: no income decline)**						
	Less than 25%	0.853	0.146	–0.930	.352	0.610-1.193
	50%	0.812	0.195	–0.870	.386	0.507-1.300
	75%	0.593	0.233	–1.330	.184	0.274-1.281
	100% (I have lost all my income)	1.078	0.350	0.230	.818	0.570-2.038
Currently unemployed		0.973	0.187	–0.140	.885	0.667-1.418
**Location (reference: urban area)**						
	Semiurban/suburban	1.421	0.205	2.430	.015	1.071-1.887
	Rural	0.955	0.166	–0.260	.792	0.680-1.342
Sex: female		1.270	0.164	1.860	.063	0.987-1.635
Live alone		1.131	0.222	0.630	.529	0.771-1.662
Have chronic illness		1.218	0.153	1.570	.117	0.952-1.559
**Deferred care in**						
	Orthopedics	0.998	0.777	0.000	.998	0.217-4.591
	Mental health	4.551	4.483	1.540	.124	0.660-31.373
	Neurology	0.788	0.778	–0.240	.809	0.114-5.454
	Pediatrics	1.328	1.380	0.270	.785	0.173-10.176
	Radiology/imaging	0.699	0.557	–0.450	.653	0.147-3.330
	Surgery	0.545	0.467	–0.710	.479	0.102-2.925
	Primary care	1.840	1.382	0.810	.417	0.422-8.023
	Cancer	0.996	0.983	0.000	.997	0.144-6.894
	Cardiovascular	0.930	1.003	–0.070	.947	0.113-7.688
	Internal medicine	1.654	1.466	0.570	.570	0.291-9.395
	Women’s health/obstetrics and gynecology	1.032	0.811	0.040	.968	0.222-4.810
	Dental services	0.538	0.401	–0.830	.406	0.124-2.321
	Other	1.005	0.758	0.010	.995	0.229-4.407

## Discussion

The COVID-19 pandemic created an immediate problem with patients being unable to access care as they normally would, but it also presented an opportunity for telemedicine. In terms of how much of a problem deferred care is, our data present a nuanced interpretation. On the one hand, nearly half the sample deferred care, suggesting that deferred care is a substantial problem. On the other hand, respondents were highly unconcerned about the health effect associated with care deferral and report that it was largely for preventive care, with preventive dental services being the top deferred service. However, there was a subgroup—about a quarter of those who deferred care—who were very concerned about the health effects of the deferral; this group was more likely to defer care in areas like surgery and orthopedics.

Telemedicine has still not been used by the majority of the sample, but respondents indicated a strongly increased willingness to try, particularly for deferred care. This effect was especially significant in older females and more educated respondents, but lower for persons who actually had deferred care. A recent review of barriers to telemedicine adoption identified the top six barriers as technically challenged staff (11%), resistance to change (8%), cost (8%), reimbursement (5%), patient age (5%), and level of education of the patient (5%) [13]. The pandemic may have loosened resistance to change, while policy changes altered cost and reimbursement issues. Our findings suggest that age may be less of a factor than suggested by previous research; this is likely due to the lack of alternatives for older persons to receive needed care. This suggests the potential for a permanent shift to telemedicine, assuming reimbursement policies remain in place.

We examined deferred care in the context of an unusual circumstance—the COVID-19–related medical care shutdown. Previous research on deferred care has tended to focus on particular locations such as the emergency room [14], specific populations such as Medicaid enrollees [15] or lower income persons [16], or even particular countries [17]. None of these examples are fully analogous to the situation reported in this paper, where widespread care deferral is seen in a higher income country. The applicability of these findings is most relevant to other high-income countries facing broad pandemic-related shutdowns.

This study has a number of potential limitations. First, the study sample is the primary care population of the Burlington area, and the study was conducted in late March / early April of 2020. The location is a semiurban area with limited racial diversity, extremely high insurance coverage, and relatively high levels of internet access and education. Generalizing to other locations, including other countries and areas that are either more or less urban should be done with caution. The survey was also (purposely) conducted during a time when usual medical care was unavailable. Whether the sentiments expressed in this survey would be true in the future is unknown. The sample also was already engaged to some extent with the health care system (at least one visit in the previous 3 years), so individuals without contact with the health care system may react differently. Finally, the sample was somewhat more educated, wealthier, and comprised more females than the overall population, with 48% of the sample reporting an income of over $100,000 and 76% having completed college. This somewhat reflects the community, which tends have both a higher income and more education than the United States as a whole [18].

Given the variation in COVID-19 impact across the United States and uncertainties about pandemic duration and potential new waves of infection, our data support potential increased use of telemedicine to support access to health care. We observed evidence of increased acceptance, particularly among older and female respondents. Mental health services had a high level of deferred ongoing care and a high willingness to resolve care deferral through telemedicine. This presents an opportunity for health systems as they prepare for a potential second wave. Additionally, our findings suggest that the pandemic may have permanently changed consumers’ willingness to use telemedicine for health care, particularly among older individuals. This newfound acceptance of telemedicine, coupled with insurers’ decision to continue reimbursing telemedicine at levels consummate with in-person care, suggest that higher levels of telemedicine in health care may be an enduring change.

## Abbreviations

OR: odds ratio
